# Comparison of fludarabine/melphalan (FM140) with fludarabine/melphalan/BCNU (FBM110) in patients with relapsed/refractory AML undergoing allogeneic hematopoietic cell transplantation – a registry study on behalf of the EBMT Acute Leukemia Working Party

**DOI:** 10.1038/s41409-024-02499-6

**Published:** 2024-12-19

**Authors:** Jesús Duque-Afonso, Jürgen Finke, Maud Ngoya, Jacques-Emmanuel Galimard, Johannes Schetelig, Matthias Eder, Wolf Rösler, Gesine Bug, Andreas Neubauer, Matthias Edinger, Gerald. G. Wulf, Pavel Jindra, Hermann Einsele, Matthias Stelljes, Dominik Selleslag, Eva Maria Wagner-Drouet, Donald Bunjes, Alexandros Spyridonidis, Eolia Brissot, Arnon Nagler, Fabio Ciceri, Mohamad Mohty

**Affiliations:** 1 Department of Hematology/Oncology, Faculty of Medicine, University of Freiburg Medical Center, Freiburg, Germany; 2 EBMT Statistical Unit, INSERM UMRs 938, Hôpital Saint Antoine, Paris, France; 3 Medical Department I, (Hematology, Oncology and Palliative Care), University Hospital Carl Gustav Carus, TU Dresden, Dresden, Germany; 4 Department of Haematology, Hemostasis, Oncology and Stem Cell Transplantation, Hannover Medical School, Hannover, Germany; 5 Department of Internal Medicine 5, University Hospital Erlangen, Erlangen, Germany; 6 Department of Medicine 2, Goethe University, Frankfurt am Main, Germany; 7 Department for Hematology/Oncology and Immunology, University Hospital Giessen and Marburg, Campus Marburg, Philipps Universität Marburg, Marburg, Germany; 8 Department of Hematology and Oncology, University of Regensburg, Regensburg, Germany; 9 Department of Hematology and Medical Oncology, University Hospital Goettingen, Goettingen, Germany; 10 Department of Hematology/Oncology, Charles University Hospital, Pilsen, Czech Republic; 11 Medical Department II, University Hospital Wuerzburg, Wuerzburg, Germany; 12 Department of Hematology/Oncology, University of Muenster, Muenster, Germany; 13 Department of Hematology, A.Z. Sint-Jan, Brugge, Belgium; 14 Department of Hematology and Oncology, University Medical Center Mainz, Mainz, Germany; 15 Department of Internal Medicine III, University Hospital Ulm, Ulm, Germany; 16 Division of Internal Medicine I, University Hospital of Patras, Patras, Greece; 17 Department of Haematology, Sorbonne University, Saint-Antoine Hospital, AP-HP, INSERM UMRs 938, Paris, France; 18 Hematology Division, Chaim Sheba Medical Center, Tel Hashomer, Israel; 19 IRCCS San Raffaele Scientific Institute, University Vita-Salute, Milan, Italy

**Keywords:** Translational research, Acute myeloid leukaemia

## Abstract

The treatment of relapsed/refractory acute myeloid leukemia (AML) is associated with a dismal prognosis. The allogeneic hematopoietic cell transplantation (allo-HCT) is frequently performed as salvage therapy. Reduced intensity conditioning protocols have been developed with the aim of reducing the leukemia burden without increasing their toxicity. We compared the reduced intensity conditioning FM140 (fludarabine, 150 mg/m^2^; melphalan 140 mg/m^2^) with FBM110 (fludarabine 150 mg/m^2^; BCNU, also known as carmustine, 300–400 mg/m^2^; and melphalan 110 mg/m^2^). From the European Bone Marrow Transplantation (EBMT) Acute Leukemia Working Party registry, we identified 293 adult patients (FM140, *n* = 118 and FBM110, *n* = 175) with AML with relapsed/refractory disease prior to allo-HCT. There were some differences such as age (FM140 = 59.5 years vs. FBM110 = 65.1 years, *p* < 0.001) and graft-versus-host disease (GvHD) prophylaxis based on in vivo T-cell depletion (TCD, FM140 = 39% vs. FBM110 = 75%, *p* < 0.001). No differences were observed between FM140- and FBM110-treated patients regarding overall survival (OS) (2-year OS: 39.3% vs. 45.7%, *p* = 0.58), progression-free survival (PFS) (2-year PFS: 36.1% vs. 37.3%, *p* = 0.69), non-relapse mortality (NRM) (2-year NRM: 15.3% vs. 25.7%, *p* = 0.10) and relapse incidence (RI) (2-year RI: 48.6% vs. 37.0%, *p* = 0.7). In conclusion, despite differences in age and GvHD prophylaxis, AML patients with active disease undergoing allo-HCT after FBM110 conditioning showed similar outcomes compared to FM140.

## Introduction

The treatment of relapsed/refractory AML is associated with a dismal prognosis [1]. The allogeneic hematopoietic cell transplantation (allo-HCT) is frequently performed as salvage therapy in this scenario [2, 3]. Conditioning protocols have been developed with the aim of reducing the leukemia burden without increasing their toxicity, especially in older patients or those with comorbidities [4].

Conditioning with fludarabine/melphalan 140 mg/m^2^ (FM140) is a standard protocol in many centers for patients with AML in complete remission [5] and in combination with sequential chemotherapy in those with active disease [6, 7]. Compared to a different commonly used protocol based also on an alkylating agent such as fludarabine/busulfan (FluBu2), overall survival (OS) appears to be similar. However, relapse incidence (RI) is lower but non-relapse mortality (NRM) is higher in patients conditioned with FM140 compared to FluBu2 [8–10]. Within conditioning protocols with a transplantation conditioning index (TCI) classified as intermediate [11, 12], we have previously shown that patients treated with FM140 have comparable outcomes to those patients treated with the single alkylator-based conditioning protocol, fludarabine/treosulfan (FluTreo) using the registry of the Acute Leukemia Working Party (ALWP) of the European Society for Blood and Marrow Transplantation (EBMT). Patients with FM140 showed a decreased RI and higher NRM resulting in similar OS [13].

In order to increase the anti-leukemic effect without increasing toxicity, conditioning with FM140 has also been modified by adding a second alkylating agent such as BCNU, also known as carmustine, and reducing the melphalan dose (FBM110) [14–16]. This conditioning protocol has been shown to have a remarkable anti-leukemic effect including patients with active AML without increasing its toxicity significantly [17, 18]. Similarly, the FTM110 conditioning protocol was established by adding the alkylator thiotepa to the FM140 backbone and reducing the melphalan dose [19]. Patients conditioned with FBM110 and FTM110 showed similar OS after adjusting for clinical variables and these protocols were suitable for older patients and those with comorbidities [20].

In our previous studies, we showed that AML patients in complete remission had better outcomes including OS after conditioning based on two alkylating agents (FBM110/FTM110) compared to single alkylator-based conditioning with FM140 [21]. In this study, using the registry of the EBMT on behalf of the ALWP, we analyzed retrospectively, outcomes of patients with active AML disease, defined as relapsed or refractory, after conditioning with the protocol FM140 compared to FBM110.

## Patient and methods

### Study design

In this retrospective multicenter analysis, data were provided by the ALWP of the EBMT, who report annually all consecutive allo-HCTs after patient authorization via informed consent. The study was approved by its general assembly. We focused on (1) adult (aged >18 years) patients who received conditioning with FM140 (fludarabine, median 150 mg/m^2^; melphalan 140 mg/m^2^) or with FBM110 (fludarabine, median 150 mg/m^2^; BCNU 300–400 mg/m^2^ and melphalan 110 mg/m^2^), (2) first allo-HCT from a matched sibling or unrelated donor for patients with (3) AML in active disease including primary induction failure (PIF), relapsed or progressive disease, (4) transplantation date between January 1st, 2009 and December 31st, 2020 (5) with an unmanipulated peripheral blood graft (no in vitro T-cell depletion (TCD) and no bone marrow grafts). Patients undergoing haploidentical allo-HCT were excluded. We excluded patients, who received high dose melphalan and fludarabine as part from sequential conditioning with f.e. high dose melphalan with FluBu2 or fludarabine/treosulfan (FluTreo) or total body irradiation/fludarabine (TBI/Flu) in order to have a cohort of patients as homogenous as possible and to avoid confounding with different substances and dosages of chemotherapies and TBI. We also excluded patients in CR2/CR3 from the analysis. Hence, we identified 68 patients conditioned with FBM140 and 5 patients with FM110, who were excluded from the analysis. As previously defined [22, 23], patients who never achieved CR despite induction and salvage chemotherapy were classified as having primary refractory AML, while patients who initially achieved CR (BM blasts ≤5%) and then experienced relapse were classified as having relapsing AML. Patients with untreated AML were excluded from the analysis. Cytogenetic risk at diagnosis was categorized according to the 2017 European Leukemia Net (ELN) recommendations for AML [1]. Six centers out of 90 used both protocols. Fifty-nine (20%) of the patients received an unrelated donor graft for which the human leucocyte antigen (HLA) matching was incomplete or had a low resolution making it impossible to calculate the high-resolution mismatches on loci A, B, C, DRB1 and DQB1. These patients were included as unrelated donors. Informed consent was obtained from all patients for use of the clinical data in research.

In contrast to our previous retrospective single-center- and multicenter-based studies [20, 21], patients conditioned with FBM110 and fludarabine, thiotepa, melphalan (FTM110) protocols showed different outcomes and so, we were not able to pool the patients in our single cohort for further analysis.

### Statistical analysis

Outcome variables were defined following internal consensus guidelines [24]. Patient-, disease- and treatment-related characteristics were compared using the chi-square test for categorical data or the Mann-Whitney test for continuous data. Baseline characteristics were summarized using median, interquartile range (IQR), and range, for continuous data, and frequency and percentage for categorical data. We assumed a normal distribution of the data and a similar variance in both groups.OS was defined as the time from allo-HCT until death from any cause. Progression-free survival (PFS) was defined as the time from allo-HCT to death from any cause, or relapse/progression, whichever occurred first. Relapse was defined as detection of disease via cytological and/or histological assessment after allo-HCT; death without prior relapse was considered as a competing risk for relapse and was denoted as NRM. For cumulative incidence of acute graft-versus-host disease (aGvHD) and chronic GvHD (cGvHD), death without aGvHD/cGvHD and relapse were considered as competing events. GvHD-free, relapse-free survival (GRFS) was defined as being alive with neither grade III-IV aGVHD nor severe cGVHD, relapse, or death from any cause post-HCT. Patients with no event were censored at the date of last follow-up. To address for the difference in follow-up period between the two conditioning regimen groups, outcome was described at 2 years post transplantation for all comparisons (no censoring at 2 years).

Univariate analyses were performed using Gray’s test for cumulative incidence functions and the log-rank test for OS, GRFS, and LFS. The Cox proportional-hazards model was used for multivariable regression analysis and included variables with unbalanced distribution between the two groups or factors known to predict outcomes. To allow for center differences, a random effect or “frailty” was introduced for each center into the models. Center effect was included in multivariate analysis. Results were expressed as the hazard ratio (HR) with the 95% confidence interval (95% CI).

All tests were two sided. The Type I error was fixed at 0.05 for factors associated with time-to-event outcomes. Statistical analyses were performed with R 4.3.2 (R Development Core Team, Vienna, Austria) software packages.

## Results

### Patient and transplant characteristics

The patient and transplant characteristics of the 293 AML patients are shown in Table 1. Patients in the FBM110 group were older (65.1 years vs. 59.5 years, *p* < 0.001), had a lower HCT-CI score (HCT-CI ≥3: 34% vs. 54%, *p* = 0.02), had more often secondary AML (35% vs. 25%, *p* = 0.049) and received more often in vivo TCD (92% vs. 78%, *p* < 0.001). Patients conditioned with FBM110 received more often in vivo TCD based on anti-thymocyte globulin (ATG, 75% vs. 39%) compared to patients conditioned with FM140, who received alemtuzumab more frequently (17% vs. 39%). Other patient and transplant characteristics such as patient sex (*p* = 0.47), donor sex (*p* = 0.42), Karnofsky performance status score (KPS) < 90 (*p* = 0.48), disease status of PIF or relapse (*p* = 0.95), cytogenetic risk group (*p* = 0.54), donor type (*p* = 0.17), neutrophile and thrombocyte engraftment (*p* = 0.87 and *p* = 0.08) did not differ between FBM110 and FM140 groups.

**Table 1:** Patient characteristics.

	FM140	FBM110	*P*-value
*N* (%)	118 (40)	175 (60)	
Melphalan dose (mg/m^2^)	140	110	
Median follow-up in years (95% CI)	5 (3.2–6.4)	5.1 (4.1–6.6)	
Median time from diagnosis to allo-HCT, in months (range)	6.7 (0.3–101.3)	5.4 (0.9–110.4)	0.35
Patient age in years, median (range)	59.5 (18.3–73.7)	65.1 (35.6–77.9)	<0.001
Patient sex, female, *n* (%)	51 (43)	83 (47)	0.47
Donor sex, female, *n* (%)	85 (29)	31 (26)	0.42
Donor sex female to patient sex male, *n* (%)	10 (8.5)	31 (18)	0.03
KPS score <90, *n* (%)	46 (42)	80 (47)	0.48
HCT-CI score, *n* (%)			0.02
- 0	27 (25)	33 (38)	
- 1–2	25 (22)	24 (28)	
- ≥3	61 (54)	29 (34)	
- missing	62	33	
AML diagnosis *n* (%)			
- de novo	89 (75)	113 (65)	0.049
- secondary AML	29 (25)	62 (35)	
Disease status *n* (%)			
- PIF	69 (58)	103 (59)	0.95
- Relapse/PD	49 (42)	72 (41)	
Cytogenetics *n* (%)			
- Favorable	4 (3)	5 (3)	0.54
- Intermediate	42 (36)	61 (35)	
- Poor	24 (20)	26 (15)	
Donor type *n* (%)			0.17
- MSD	30 (25)	33 (19)	
- UD	88 (75)	142 (81)	
GvHD prophylaxis *n* (%)			<0.001
- CsA	47 (40)	26 (15)	
- CsA + MMF	33 (28)	112 (64)	
- CsA + MTX	26 (22)	15 (9)	
- MMF + Tacrolimus	7 (6)	8 (5)	
- Other	5 (4)	14 (8)	
In vivo TCD *n* (%)			
- no in vivo TCD	26 (22)	14 (8)	<0.001^a^
- in vivo TCD	92 (78)	161 (92)	
- ATG	−46 (39)	−132 (75)	<0.001^b^
- Alemtuzumab	−46 (39)	−29 (17)	
Engraftment, %, (95% CI)			
- Neutrophiles recovery (30d)	89 (82–94)	94 (89–97)	0.87
- Neutrophiles recovery (60d)	92 (85–96)	96 (91–98)	
- Thrombocytes >20Tsd/ul (30d)	85 (76–91)	83 (75–89)	0.08
- Thrombocytes >20Tsd/ul (60d)	89 (80–94)	93 (87–97)	

*KPS* Karnofsky performance status, *AML* acute myeloid leukemia, *PIF* primary induction failure, *PD* progressive disease, *MSD* matched sibling donor, *UD* unrelated donor, *GvHD* graft-versus-host disease, *CsA* cyclosporine A, *MTX* methotrexate, *MMF* mycophenolate mofetil, *TCD* T-cell depletion, *ATG* anti-thymocyte globulin, *IQR* interquartile range, *CI* confidence interval.

^a^Statistical difference between with and without in vivo TCD.

^b^Statistical difference between ATG vs alemtuzumab.

### Analysis of outcomes in patients conditioned with FM140 compared to FBM110

#### OS, PFS, RI and NRM

**Fig. 1:**
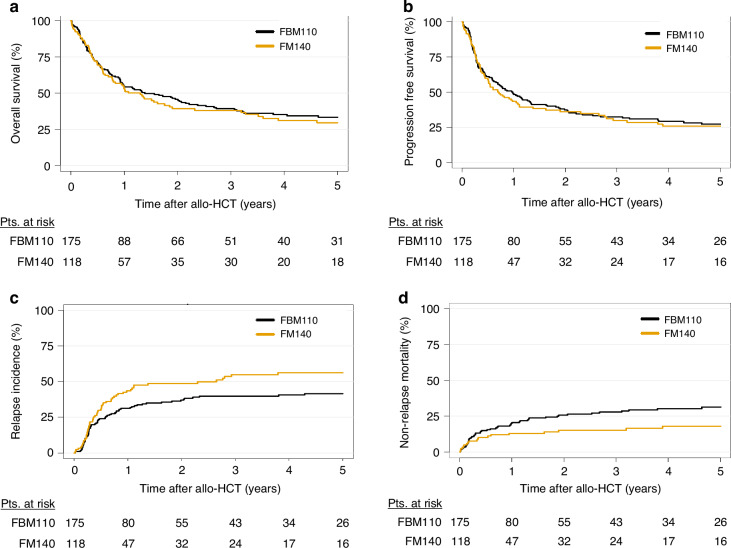
Impact of conditioning by FM140 and FBM110 on outcome. Kaplan–Meier curves represent (**a**) overall survival and **b** progression-free survival. Cumulative incidences of **c** relapse and **d** non-relapse mortality. FM140 fludarabine/melphalan, FBM110 fludarabine/BCNU/melphalan, Pts. patients, allo-HCT allogeneic hematopoietic cell transplantation.

We compared FM140 with FBM110 conditioning in univariate (Table 2, Fig. 1) and multivariate analysis (Table 3). According to multivariate analysis of outcome variables at 2 years, no statistically significant differences were observed between FBM110- compared to FM140-treated patients regarding OS (FBM110 vs. FM140, 45.7% vs. 39.3%, HR in multivariate analysis for FM140 = 0.9, *p* = 0.58), PFS (37.3% vs 36.1%, HR = 0.93, *p* = 0.69), NRM 25.7% vs 15.3%, HR = 0.59, *p* = 0.1) and RI (37% vs. 48.6%, HR = 0.89, *p* = 0.7).

**Table 2:** Estimate incidence for outcome variables according to conditioning protocol.

Outcomes*	FM140	FBM110
*n* (%)	118 (40)	175 (60)
OS at 2 y	39.3 (29.9–48.5)	45.7 (37.9–53.2)
PFS at 2 y	36.1 (27.1–45.1)	37.3 (29.9–44.7)
RI at 2 y	48.6 (38.9–57.6)	37 (29.6–44.3)
NRM at 2 y	15.3 (9.3–22.8)	25.7 (19.3–32.6)
aGvHD II/IV at 100 d	21.6 (14.3–29.9)	36.6 (29.5–43.8)
aGvHD III/IV at 100 d	7.5 (3.5–13.6)	15.1 (10.2–20.9)
GRFS at 2 y	27 (18.9–35.8)	25.1 (18.7–32.0)
cGvHD at 2 y	28.2 (19.7–37.3)	30.8 (23.7–38)
cGvHD ext. at 2 y	7.9 (3.4–14.7)	17 (11.6–23.2)

*OS* overall survival, *PFS* progression-free survival, *RI* relapse incidence, *NRM* non-relapse mortality, *aGvHD* acute graft-versus-host disease, *cGvHD* chronic graft-versus-host disease, *Ext* extensive, *GRFS* GvHD-/relapse-free survival, *FluMel* fludarabine/melphalan, *FBM* fludarabine/BCNU/melphalan, *CI* confidence interval, *d* day, *y* year.

*Outcomes were described at 2 years except aGvHD, which was described at 100 days.

**Table 3:** Multivariate analysis of outcome variables.

	Overall survival		Progression-free survival		Relapse		NRM	
	HR (95% CI)	*p*-value	HR (95% CI)	*p*-value	HR (95% CI)	*p*-value	HR (95% CI)	*p*-value
Conditioning Regimen FBM110 vs FM140	0.9 (0.63–1.3)	0.58	0.93 (0.64–1.35)	0.69	0.89 (0.5–1.59)	0.7	0.59 (0.32–1.11)	0.1
Year of allo-HCT	0.95 (0.9–1.00)	0.04	0.96 (0.91–1.01)	0.12	0.94 (0.88 – 1.01)	0.08	0.99 (0.91–1.08)	0.82
Increasing age at allo-HCT by 10 y.	0.84 (0.71–1)	0.046	0.89 (0.76–1.06)	0.18	0.87 (0.7–1.08)	0.2	0.88 (0.65–1.19)	0.42
Female donor to male patient	0.88 (0.57–1.35)	0.56	0.85 (0.56–1.3)	0.45	0.81 (0.47–1.4)	0.44	0.8 (0.4–1.59)	0.52
Unrelated donor	1.13 (0.74–1.73)	0.57	1.18 (0.77–1.79)	0.45	1.08 (0.62–1.88)	0.77	1.26 (0.62–2.56)	0.52
CMV donor positive	0.66 (0.46–0.94)	0.02	0.76 (0.54–1.07)	0.12	1.05 (0.68–1.63)	0.82	0.49 (0.28–0.85)	0.01
CMV patient positive	1.54 (1.07–2.23)	0.02	1.35 (0.95– 1.92)	0.09	1.12 (0.7–1.78)	0.64	1.82 (1.04–3.18)	0.04
PIF vs Relapse/PD	1.35 (0.99–1.85)	0.057	1.3 (0.96–1.76)	0.09	1.26 (0.86–1.84)	0.24	1.37 (0.81–2.31)	0.24
In vivo T-cell depletion	0.46 (0.28–0.77)	0.003	0.48 (0.29– 0.79)	0.004	0.54 (0.28–1.03)	0.06	0.42 (0.17–1.01)	0.052
Cytogenetics good/interm. vs. poor	0.95 (0.66–1.35)	0.76	1 (0.71–1.42)	0.99	1.02 (0.63–1.65)	0.93	1.12 (0.63–1.97)	0.71
Cytogenetics good/interm. vs. NA/failed	1.34 (0.86–2.08)	0.2	1.22 (0.79–1.87)	0.37	1.78 (1.05 – 3.01)	0.03	0.62 (0.25 –1.52)	0.29
Secondary AML	1.12 (0.8–1.56)	0.52	0.96 (0.7–1.32)	0.8	0.71 (0.46–1.09)	0.12	1.44 (0.87–2.37)	0.16
KPS < 90	1.52 (1.11–2.08)	0.009	1.44 (1.07–1.95)	0.02	1.05 (0.7–1.57)	0.82	2.29 (1.39–3.77)	0.001

A Center effect or “frailty” was included in the model. Year of allo-HCT was included as an integer and not as continuous variable. The HR of year of allo-HCT was calculated corresponding to an increase of 3 years.

*FM140* fludarabine/melphalan, *FBM110* fludarabine/BCNU/melphalan, *allo-HCT* allogeneic hematopoietic cell transplantation, *NRM* non-relapse mortality, *HR* hazard ratio, *CI* confidence interval, *AML* acute myeloid leukemia, *KPS* Karnofsky performance status, *CMV* cytomegalovirus, *NA* not assessed, *PIF* primary induction failure, *PD* progressive disease.

#### GvHD and GRFS

Results regarding GvHD severity and incidence are shown in Tables 2, 3, and Supplementary Table 1. There were no statistically significant differences in 100-day incidence of aGvHD grades II-IV compared to FM140 patients (36.6% vs. 21.6%, HR in multivariate analysis for FM140 = 0.66, *p* = 0.19), or in 100-day aGvHD grades III-IV (aGvHD III-IV: 15.1% vs. 7.5%, HR = 0.67, *p* = 0.35) or in 2-year cGvHD (30.8% vs. 28.2%, HR = 0.88, *p* = 0.53). GFRS at 2 years was also similar between the treatment groups (25.1% vs. 27%, HR 0.8, *p* = 0.16).

### Other variables associated with outcomes

#### OS, PFS, RI and NRM

We observed that the increment in each year of transplant was positively associated with OS (HR 0.95, *p* = 0.04) and increasing age by 10 years at transplant was shown to be a favorable factor for OS (HR 0.84, *p* = 0.046). KPS < 90 was an unfavorable prognostic factor for OS (HR 1.52, *p* = 0.009) as well as for PFS (HR = 1.44, *p* = 0.02) and NRM (HR 2.29 *p* < 0.001). Interestingly, positive CMV status for donor was shown to be a favorable factor for OS and NRM (HR 0.66, *p* = 0.02; HR 0.49, *p* = 0.01, respectively), and patient CMV positivity was an unfavorable factor for OS and NRM (HR 1.54, *p* = 0.02; HR 1.82, *p* = 0.04, respectively). Use of in vivo TCD as GvHD prophylaxis was shown to be a favorable factor for OS (HR 0.46, *p* = 0.003) and PFS (HR 0.48 *p* = 0.004) (Table 3).

#### GvHD and GRFS

Increasing age by 10 years at transplant was a favorable factor for cGVHD (HR 0.78, *p* = 0.008) as was in vivo TCD for cGvHD and GFRS (HR 0.53, *p* = 0.04; HR = 0.4 *p* = 0.001, respectively). KPS < 90 was associated with increased risk of cGVHD (HR 1.44, *p* = 0.04) and GRFS (HR 1.53, *p* = 0.003) (Supplementary Table 1).

#### Cause of death

Cause of death in patients undergoing allo-HCT conditioned with FM140 compared to FBM110 is described in Supplementary Table 2. Most patients died due to underlying disease (FBM110, *n* = 58 [53%]; FM140: *n* = 55 [73%]). Other frequent causes of death were infections (FBM110 *n* = 19 [17%]; FM140 *n* = 6 [8%]) and GvHD (FBM110 *n* = 13 [12%]; FM140 *n* = 4 [5%]), which were numerically different between the groups.

## Discussion

Despite the recent improvements in AML therapies, PIF with relapsed/refractory AML is still clinically challenging and remains an unmet clinical need [25]. Targeted therapies for specific subgroups as well as salvage chemotherapy play a role as bridging therapy to control or reduce AML activity. Nevertheless, an allo-HCT is the only potentially curative option in this scenario.

Modification of conditioning regimens before allo-HCT is a strategy to improve outcome of AML patients with active disease. Sequential chemotherapy with FLAMSA (fludarabine, amsacrine, cytarabine) [26] or TEC (thiotepa, etoposide, cyclophosphamide) [27] as induction chemotherapy followed shortly by a reduced toxicity conditioning has been established as salvage chemotherapy prior to allo-HCT. A randomized clinical trial has recently shown the non-inferiority of undergoing immediately to allo-HCT compared to receiving a high-dose salvage chemotherapy prior to allo-HCT in patients with active AML disease, suggesting patients with relapsed/refractory AML should proceed to allo-HCT as soon as possible [28].

Hence, modifying conditioning chemotherapy prior to allo-HCT has been performed in order to improve outcome of AML patients. Examples of strategies to optimize conditioning therapy include exchanging alkylating chemotherapy with treosulfan [29], adding total body irradiation to sequential chemotherapy conditioning [7, 30] or adding novel therapies such as venetoclax [31] or decitabine [32].

In this retrospective study, we focused on analyzing the outcomes of patients with AML with active disease, including PIF and relapsed/refractory disease, after conditioning with the FBM110 protocol containing two alkylating agents (BCNU and melphalan, FBM110) compared to the backbone protocol with one alkylating agent (melphalan, FM140). Due to the retrospective nature of the study, clinical characteristics were unbalanced between the conditioning groups. Patients conditioned with FBM110 were significantly older, had lower HCT-CI score, suffered more often of secondary AML and received more often in vivo TCD. Hence, the latter patients received more frequently ATG and FM140 patients received alemtuzumab as in vivo TCD.

Although patients conditioned with FBM110 showed numerically less RI at 2 years (48.6% vs. 37%) and increased NRM (25.7% vs. 15.3%), these differences were not statistically significant in multivariate analysis. Therefore, we concluded that despite differences in clinical characteristics such as age and GvHD prophylaxis, patients conditioned with FBM110 or FM140 showed similar outcomes in OS, PFS, RI and NRM. Here, we speculate that the graft vs leukemia effect seems to be independent of a second alkylating agent. Hence, the incidence of aGvHD, cGVHD and GFRS was also similar between both conditioning groups.

Interestingly, patients with a ten year increase in age have significantly better overall survival in multivariate analysis (Table 3, HR 0.84, CI 0.71-1, *p* = 0.046). Although counterintuitive at first, we speculate that younger patients, not fit for a myeloablative conditioning, receive a conditioning with a TCI intermediate score as FM140 or FBM110. These younger unfit patients have a worse overall survival that older fit patients, who receive standard FM140 or FBM110, suggesting that fitness including co-morbidities and Karnofsky performance score are so, or even more, important than age determining outcome, as recently suggested [33]. We also speculate that the older patients might have received novel therapies as hypomethylating agents in combination with venetoclax, which preserve their fitness prior allo-HCT [34, 35]. In contrast, to younger patients which might have received more toxic induction chemotherapy regimens prior allo-HCT.

Despite not observing statistically significant differences between patients conditioned with FBM110 compared to FM140, it is remarkable that about 45% in the FBM110 group and 40% of the patients in the FM140 group are alive after 2 years and about 30% of the patients in each group are still alive 5 years post allo-HCT. This indicates allo-HCT as an effective therapy in patients with relapsed/refractory AML even at advanced age or with co-morbidities that should be considered in this unfavorable condition.

Donors with CMV status positive were associated with an improved OS and reduced NRM. Furthermore, patients with CMV status positive were associated with a decreased OS and increased NRM. We speculate that donors with CMV donor positive have already immunity against CMV and transplanted patients have less CMV reactivation after alloHCT. Future studies should analyze, if this observation is still currently valid in the era of CMV prophylaxis with letermovir in high risk patients for CMV reactivation after allo-HCT.

This study has some limitations. First, this is a retrospective study of patients included in the EBMT ALWP registry from several centers, mostly in Europe. The gold standard in order to compare two different conditioning regimens is the randomized controlled trial. However, such studies are very expensive to conduct and are very time- and resource-consuming. Second, It is possible, that our statistical analysis did not have enough power to detect subtle differences due to the number of patients (*n* = 293). Third, the two cohorts had unbalanced patient characteristics e.g. age, and GvHD prophylaxis regimens, which might have influenced outcomes. The EBMT ALWP registry does not have data about % of blasts and mutation profile, which might have differed between the groups. Finally, both protocols are center- and country-specific. FM140 was used more frequently in Great Britain (*n* = 43, 36%), Germany (*n* = 28, 24%) and Belgium (*n* = 21, 18%) and FBM110 was used almost exclusively in Germany (*n* = 170, 97%). This might account for differences in country-specific transplantation practices not described in the data collected by the EBMT.

Our results show that intensification of chemotherapy-based conditioning regimens with the addition of a second alkylating chemotherapy (BCNU) to melphalan in a dosage of 110 mg/m^2^ (FBM110) prior to allo-HCT is highly efficient but may not further improve the outcome of patients with AML in active disease compared to melphalan in a dosage of 140 mg/m^2^ (FM140). To which extent other combination partners (f.e. thiotepa) with melphalan bring an advantage remains speculative. Future strategies for the treatment of patients with relapsed/refractory AML should include novel strategies prior to and after allo-HCT, including targeted therapies, GvHD prophylaxis, novel conditioning protocols and prophylactic infusions of donor lymphocytes. Alternative strategies focused on maintenance therapy after allo-HCT (measurable residual disease-guided) with targeted therapies [36] as well as use of hypomethylating agents with or without venetoclax [37–40], specific antibodies [41, 42] should also be considered.

In conclusion, despite differences in age, HCT-CI score, frequency in sAML and GvHD prophylaxis based on in vivo TCD, AML patients with relapsed/refractory disease undergoing allo-HCT after FBM110 and FM140 conditioning show similar outcomes. We speculate that the reduced dose of melphalan is compensated by the addition of the second alkylating agent (BCNU) in FBM110 without increasing significantly toxicity or RI. Allo-HCT with toxicity-reduced conditioning is an effective therapy for older patients or those with comorbidities, with relapsed or chemotherapy refractory AML, and it should be considered in this patient population. Future studies should address the efficacy of melphalan-containing regimens in combination with other drugs and/or use of different dosing.

## Supplementary information

Supplementary material

## Data Availability

The datasets and computer codes generated during and/or analyzed during the current study are available upon reasonable request from the corresponding authors.
