# Supplementary material for: Comparison of fludarabine/melphalan (FM140) with fludarabine/melphalan/BCNU (FBM110) in patients with relapsed/refractory AML undergoing allogeneic hematopoietic cell transplantation – a registry study on behalf of the EBMT Acute Leukemia Working Party
Source: Bone Marrow Transplant. 2024 Dec 19;60(3):373–9. doi: 10.1038/s41409-024-02499-6 (PMC11893469; doi:10.1038/s41409-024-02499-6)
Supplement: Supplementary file 1 — Supplementary material [file 41409_2024_2499_MOESM1_ESM.docx]

|  | **aGvHD II-IV** | | **aGvHD III-IV** | | **cGvHD** | | **GRFS** | |
| --- | --- | --- | --- | --- | --- | --- | --- | --- |
|  | **HR (95% CI)** | **p-value** | **HR (95% CI)** | **p-value** | **HR (95% CI)** | **p-value** | **HR (95% CI)** | **p-value** |
| **Conditioning Regimen FBM110 vs FM140** | 0.66  (0.35-1.22) | 0.19 | 0.67  0.28 – 1.56) | 0.35 | 0.88  (0.59-1.32) | 0.53 | 0.8  (0.58-1.1) | 0.16 |
| **Year of allo-HCT** | 1.06  (0.97-1.15) | 0.18 | 1.1  (0.97-1.25) | 0.14 | 1.0  (0.94 – 1.06) | 0.95 | 0.97  (0.93-1.02) | 0.27 |
| **Age at allo-HCT by 10y.** | 1.1  (0.82-1.48) | 0.52 | 1.15  (0.72-1.85) | 0.56 | 0.78  (0.65-0.94) | 0.008 | 0.9  (0.77-1.05) | 0.16 |
| **Female donor to male patient** | 1.23  (0.69-2.21) | 0.48 | 1.59  (0.7 -3.63) | 0.27 | 1.27  (0.77-2.07) | 0.35 | 0.78  (0.52-1.17) | 0.23 |
| **Unrelated donor** | 1.23  (0.63-2.39) | 0.55 | 1.74  (0.57 – 5.26) | 0.33 | 0.97  (0.57-1.63) | 0.91 | 0.95  (0.66-1.38) | 0.8 |
| **CMV donor positive** | 0.78  (0.47-1.31) | 0.36 | 1.18  (0.56-2.52) | 0.66 | 0.78  (0.52-1.17) | 0.23 | 0.79  (0.57-1.08) | 0.14 |
| **CMV patient positive** | 1.12  (0.66-1.89) | 0.67 | 1.46  (0.65- 3.28) | 0.36 | 1.2  (0.79-1.82) | 0.4 | 1.38  (1-1.92) | 0.052 |
| **PIF vs relapse/PD** | 0.97  (0.6-1.54) | 0.88 | 1.27  (0.62-2.61) | 0.51 | 1.14  (0.78-1.65) | 0.5 | 1.02  (0.77-1.35) | 0.9 |
| ***In vivo* T-cell depletion** | 1.04  (0.28-0.77) | 0.93 | 0.62  (0.18- 2.14) | 0.45 | 0.53  (0.29-0.96) | 0.04 | 0.4  (0.25-0.62) | <0.001 |
| **Cytogenetics good/interm. vs. poor** | 1.22  (0.67-2.22) | 0.52 | 2.36  (0.98-5.64) | 0.056 | 1.12  (0.72-1.73) | 0.62 | 1.02  (0.74-1.42) | 0.89 |
| **Cytogenetics good/interm. vs. NA/failed** | 1.62  (0.86 – 3.04) | 0.14 | 1.15  (0.39 – 3.4) | 0.79 | 1.55  (0.95 – 2.53) | 0.08 | 0.94  (0.62-1.42) | 0.77 |
| **Secondary AML** | 0.89  (0.55 - 1.45) | 0.65 | 1.63  (0.81-3.29) | 0.17 | 0.84  (0.57-1.25) | 0.39 | 0.93  (0.69-1.25) | 0.63 |
| **KPS <90** | 1.48 (0.94-2.35) | 0.09 | 1.74 (0.87-3.48) | 0.12 | 1.44 (1.02-2.04) | 0.04 | 1.53  (1.16-2.03) | 0.003 |

**Supplementary Table 1**

**Supplementary Table 1. Multivariate analysis of outcome variables.** FM140, fludarabine/melphalan; FBM110, fludarabine/BCNU/melphalan; aGvHD, acute graft-versus-host disease; cGvHD, chronic graft-versus-host disease; GRFS, GvHD-/relapse-free survival; HR, hazard ratio; CI, confidence interval; AML, acute myeloid leukemia; KPS, Karnofsky performance status; CMV, cytomegalovirus; NA, not assessed; PIF, primary induction failure; PD, progressive disease; A Center effect or “frailty” was included in the model. Year of allo-HCT was included as an integer and not as continuous variable. The HR of year of allo-HCT was calculated corresponding to an increase of 3 years. Patients were censored at 2 years of follow up.

**Supplementary Table 2**

|  | **FM140** | **FBM110** |
| --- | --- | --- |
| Total number of patients | 118 | 175 |
| Deaths, n (%) | 75 (64) | 113 (65) |
| Progress/relapse, n (%) | 55 (73) | 58 (53) |
| Infection, n (%) | 6 (8) | 19 (17) |
| GvHD, n (%) | 4 (5) | 13 (12) |
| Allo-HCT related, n (%) | 5 (7) | 10 (9) |
| Other, n (%) | 3 (4) | 8 (7) |
| Secondary malignancy, n (%) | 2 (3) | 2 (2) |
| Missing, n (%) | 0 | 3 (2) |

**Supplementary Table 2. Cause of death according to conditioning protocol.** FM140, fludarabine/melphalan; FBM110, fludarabine/BCNU/melphalan; GvHD, graft-versus-host disease; allo-HCT, allogeneic hematopoietic cell transplantation.
